# Tumor hypoxia induces nuclear paraspeckle formation through HIF-2α dependent transcriptional activation of NEAT1 leading to cancer cell survival

**DOI:** 10.1038/onc.2014.378

**Published:** 2014-11-24

**Authors:** H Choudhry, A Albukhari, M Morotti, S Haider, D Moralli, J Smythies, J Schödel, C M Green, C Camps, F Buffa, P Ratcliffe, J Ragoussis, A L Harris, D R Mole

**Affiliations:** 1 Department of Biochemistry, Faculty of Science, King Abdulaziz University, Jeddah, Saudi Arabia; 2 The Wellcome Trust Centre for Human Genetics, University of Oxford, Headington, Oxford, UK; 3 Weatherall Institute of Molecular Medicine, Department of Oncology, University of Oxford, Oxford, UK; 4 The Henry Wellcome Building for Molecular Physiology, University of Oxford, Headington, Oxford, UK; 5 Department of Nephrology and Hypertension, Friedrich-Alexander-University Erlangen-Nuremberg, Erlangen, Germany; 6 Old Road Campus Research Building, University of Oxford, Headington, Oxford, UK; 7 McGill University and Genome Quebec Innovation Centre, Montreal, Quebec, Canada; 8 BSRC Alexander Fleming, Athens, Greece

## Abstract

Activation of cellular transcriptional responses, mediated by hypoxia-inducible factor (HIF), is common in many types of cancer, and generally confers a poor prognosis. Known to induce many hundreds of protein-coding genes, HIF has also recently been shown to be a key regulator of the non-coding transcriptional response. Here, we show that NEAT1 long non-coding RNA (lncRNA) is a direct transcriptional target of HIF in many breast cancer cell lines and in solid tumors. Unlike previously described lncRNAs, NEAT1 is regulated principally by HIF-2 rather than by HIF-1. NEAT1 is a nuclear lncRNA that is an essential structural component of paraspeckles and the hypoxic induction of NEAT1 induces paraspeckle formation in a manner that is dependent upon both NEAT1 and on HIF-2. Paraspeckles are multifunction nuclear structures that sequester transcriptionally active proteins as well as RNA transcripts that have been subjected to adenosine-to-inosine (A-to-I) editing. We show that the nuclear retention of one such transcript, F11R (also known as junctional adhesion molecule 1, JAM1), in hypoxia is dependent upon the hypoxic increase in NEAT1, thereby conferring a novel mechanism of HIF-dependent gene regulation. Induction of NEAT1 in hypoxia also leads to accelerated cellular proliferation, improved clonogenic survival and reduced apoptosis, all of which are hallmarks of increased tumorigenesis. Furthermore, in patients with breast cancer, high tumor NEAT1 expression correlates with poor survival. Taken together, these results indicate a new role for HIF transcriptional pathways in the regulation of nuclear structure and that this contributes to the pro-tumorigenic hypoxia-phenotype in breast cancer.

## Introduction

Activation of hypoxia pathways is a common feature of many types of cancer and frequently correlates with an aggressive tumor phenotype and adverse clinical outcome.^1^ It may arise either from the hypoxic tumor microenvironment, or as a direct result of oncogenic activation or tumor suppressor inactivation. A major mechanism mediating oxygen-dependent transcriptional responses is hypoxia-inducible factor (HIF). HIF is a family of heterodimeric transcription factors comprising a common, constitutive HIF-1β subunit and a regulated HIF-α subunit.^2^ HIF-1 contains a HIF-1α subunit and HIF-2 contains a HIF-2α subunit each complexed with HIF-1β. HIF controls the expression of many hundreds of genes with important roles in oncogenic pathways including the regulation of proliferation, apoptosis, tumor metabolism, epithelial-to-mesenchymal transition, invasiveness and pH regulation.^3^ To date, study has largely focused on the regulation of protein-coding genes by these pathways.^4^ However, new sequencing technologies are identifying increasing numbers of non-coding transcripts with regulatory roles that are also important in cancer biology.^5, 6^ Pangenomic studies have shown that many of these non-coding genes are also regulated by hypoxia and that long non-coding RNAs (lncRNAs), in particular, are regulated by HIF transcriptional pathways.^5^ In addition, several studies have demonstrated the regulation of specific lncRNAs in hypoxia, including H19,^7^ lncRNA-low expression in tumor,^8^ lincRNA-p21,^9^ hypoxia-induced noncoding ultra-conserved transcripts,^10^ Linc-RoR^11^ and urothelial carcinoma-associated 1 (UCA1)^12^ many of which have important roles in cancer.

One of the most highly regulated lncRNAs in the recent pangenomic datasets was nuclear paraspeckle assembly transcript 1 (NEAT1).^5^ NEAT1 is transcribed from the familial tumor syndrome multiple endocrine neoplasia (MEN) type 1 locus on chromosome 11 and lacks any introns. The gene gives rise to two transcripts, NEAT1-1 and NEAT1-2, also called MENβ and MENɛ, which are transcribed from the same promoter, and are produced through alternate 3′-end processing.^13^ Both transcripts are nuclear in localization and are exceptionally abundant for lncRNAs. NEAT1-1 is the more abundant transcript, is approximately 3.7 kb in length and is polyadenylated.^14^ NEAT1-2 is about 23 kb long and its 3′-tail is cleaved off by RNAse P to leave a triple helical remnant that is critical for its stability.^15^ Both NEAT1-1 and NEAT1-2 are found in nuclear structures called paraspeckles.

Like cytoplasmic organelles, the nucleus is also compartmentalized, although these nuclear structures are not separated by lipid membranes. To date, little is known about how these compartments behave in hypoxia and how this might influence hypoxic gene expression. As many as 10 different types of nuclear compartments are now recognized,^16^ with paraspeckles, which form in close association with speckles, being among the most recently identified.^17^ Paraspeckles are restricted to mammalian nuclei, but are absent from embryonic stem cells. They were initially defined as foci rich in four RNA-binding proteins of the Drosophila behavior and human splicing (DBHS) family, namely RNA binding motif protein 14 (RBM14), paraspeckle component 1 (PSPC1), non-POU domain containing, octamer binding protein (NONO or p54nrb), and splicing factor proline/glutamine rich protein (SFPQ). More recently, as many as 40 paraspeckle-associated proteins have been identified of which 30 contain RNA recognition motifs and paraspeckles are rich in RNA.^14^ Both NEAT1-1 and NEAT1-2 directly interact with these proteins, are architectural components of nuclear paraspeckles, with NEAT1-2 being absolutely required for their formation.^15, 18, 19, 20^

The precise function of nuclear paraspeckles remains unclear. However, they have been shown to have at least two, not necessarily exclusive, roles in regulating gene expression. Firstly, sequestration of multifunctional protein components in paraspeckles can deplete their levels and inhibit their activity in the nucleoplasm.^21, 22^ Secondly, RNA-binding paraspeckle proteins can bind transcripts that have been subjected to A-to-I editing within Alu repeat elements, retaining them in the nucleus and potentially inhibiting their translation.^23, 24^

Here, we show the regulation of NEAT1 by hypoxia and demonstrate its generality across breast cancer cell lines and tumor models. We show that in hypoxia, NEAT1 is primarily induced by HIF-2. Hypoxia-induced NEAT1 is present in nuclear paraspeckles and induces their formation in hypoxia. Hypoxic induction of NEAT1 in turn leads to the retention of F11 receptor (F11R) RNA within the nucleus. Finally, we show that hypoxia-induced NEAT1 accelerates tumor cell proliferation and inhibits apoptosis and that high levels of tumor NEAT1 are associated with adverse clinical outcome in breast cancer.

## Results

### NEAT1 is transcriptionally regulated by HIF-2

**Figure 1:**
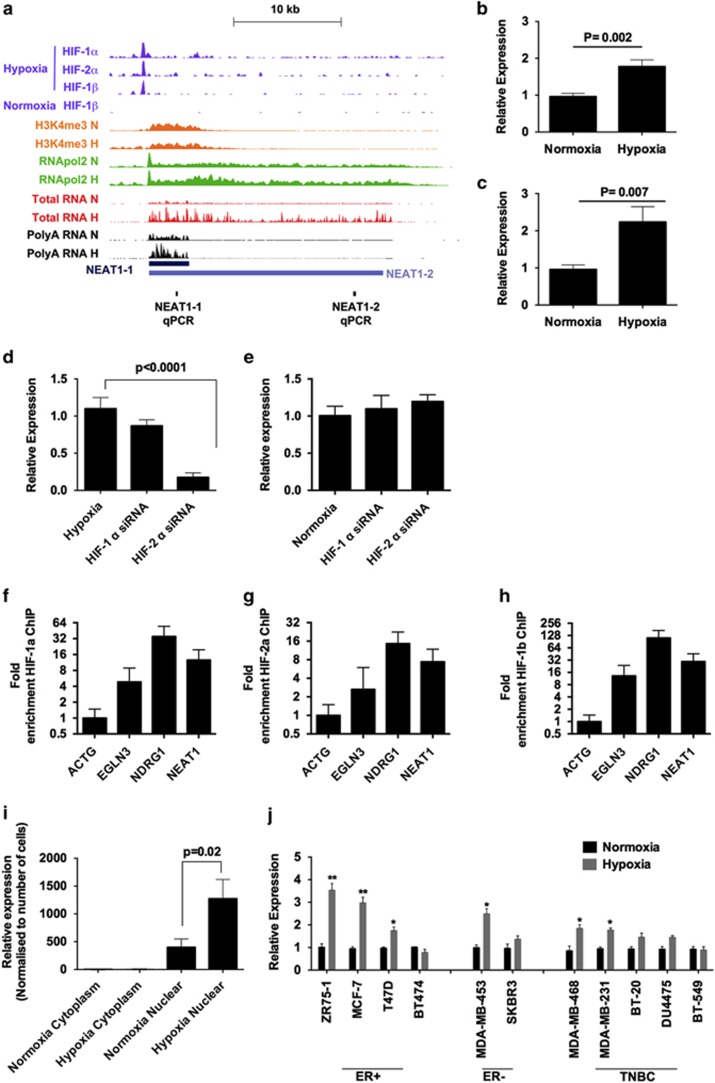
NEAT1 is transcriptionally regulated by HIF-2. (**a**) ChIP-seq and RNA-seq tracks from MCF-7 cells at the NEAT1 locus showing hypoxic induction of poly-adenylated NEAT1-1 and non-poly-adenylated NEAT1-2 transcripts. The NEAT1 promoter is enriched for H3K4me3, which increases in hypoxia. RNApol2 also binds to the promoter, with increased signal across the gene body in hypoxia. HIF-1α, HIF-2α and HIF-1β bind immediately upstream of the NEAT1 promoter. qPCR analysis of (**b**) NEAT1-1 and (**c**) NEAT1-2 transcripts in MCF-7 cells showing hypoxic upregulation of both isoforms. (**d**) qPCR analysis of the hypoxic expression of total NEAT1 in MCF-7 cells following transfection with control siRNA, HIF-1α siRNA or HIF-2α siRNA, demonstrating marked dependence on HIF-2α. (**e**) The same experiment performed in normoxia. ChIP-qPCR analysis of the NEAT1 binding site compared with ACTG negative control and EGLN3 and NDRG1 positive control sites using antibodies directed against (**f**) HIF-1α, (**g**) HIF-2α and (**h**) HIF-1β. (**i**) qPCR analysis of nuclear and cytoplasmic NEAT1 in MCF-7 cells showing that hypoxia-induced NEAT1 is expressed in the nucleus. (**j**) qPCR analysis showing hypoxic induction of NEAT1 expression across a range of estrogen receptor-positive (ER+), negative (ER-) and triple-receptor negative breast cancer cell lines (*P<0.05, **P<0.01, Student's *t*-test).

A recent pangenomic analysis of hypoxic gene regulation, in MCF-7 breast cancer cells, identified NEAT1 as one of the most hypoxia-upregulated lncRNAs.^5^ Closer inspection of these data indicated that both the 3.7-kb poly-adenylated NEAT1-1 and the 23-kb non-adenylated NEAT1-2 are upregulated by hypoxia (Figure 1a). Binding of HIF-1α, HIF-2α and HIF-1β subunits of HIF, the major transcriptional regulator of cellular responses to hypoxia was observed just upstream of the NEAT1 promoter and strongly suggests direct transcriptional control of NEAT1 by HIF. Furthermore, concomitant hypoxia-induced increases in ChIP-seq signal for RNApol2 across the NEAT1 gene body and for H3K4me3 closer to the promoter both imply transcriptional rather than posttranscriptional regulation of RNA levels by hypoxia.

We first confirmed the hypoxic induction of both NEAT1-1 and NEAT1-2 isoforms in MCF-7 cells. cDNA was generated using oligo-dT primers to amplify the polyadenylated NEAT1-1, but not NEAT1-2. This was analyzed by quantitative PCR (qPCR) using 5′-primers within NEAT1-1 confirming hypoxic regulation of NEAT1-1 (Figure 1b). Secondly, cDNA was generated using random primers, which would amplify both NEAT1 isoforms. This was analyzed by qPCR using 3′-primers within NEAT1-2, but not included in the NEAT1-1 transcript, confirming regulation of the longer isoform as well (Figure 1c). Time course analysis of total NEAT1 (NEAT1-1 and NEAT1-2) RNA levels indicated maximal induction of NEAT1 after 24 h exposure to 1% hypoxia (Supplementary Figures 1A and 1B). Treatment of cells with dimethyloxalylglycine, a non-specific inhibitor of 2-oxoglutarate dioxygenases that induces HIF by inhibition of hydroxylation, thus mimicking hypoxia, also increased NEAT1 levels, potentially implicating HIF in the regulation of NEAT1 (Supplementary Figures 1C and 1D). To test this, we pre-treated MCF-7 cells with siRNA directed against either HIF-1α or HIF-2α, to suppress each isoform, before incubating the cells in 1% hypoxia for 24 h (Supplementary Figure 2). Treatment with HIF-2α siRNA attenuated the hypoxic levels of NEAT1, whereas the effect of HIF-1α suppression was not statistically significant (Figure 1d). We also confirmed the binding of HIF-1α, HIF-2α and HIF-1β at the binding site identified by the ChIP-seq experiments using ChIP-qPCR (Figures 1f and h). Thus, although both HIF-1α and HIF-2α bind at the NEAT1 promoter, transcription is mainly regulated by HIF-2. This post-binding transcriptional selectivity is common among HIF-regulated genes.^25, 26, 27^ However, in contradistinction to these findings and to our previous pan-genomic analyses,^5^ other hypoxia-regulated lncRNAs so far described have been predominantly regulated by HIF-1.^7, 9^

Although regulated by hypoxia, NEAT1 is expressed at low level in normoxia. We therefore determined whether low levels of normoxic HIF might drive this basal NEAT1 expression. Examination of HIF-1β ChIP-seq signals for (the dimerization partner of both HIF-1α and HIF-2α) did not show any binding at the NEAT1 locus in normoxia (Figure 1a). Furthermore, suppression of neither HIF-1α nor HIF-2α had any significant effect on the expression of NEAT1 in normoxia (Figure 1e). Taken together, this indicates that normoxic NEAT1 expression is driven by additional, as yet unidentified, non-HIF pathways.

NEAT1 belongs to a class of lncRNAs that have regulatory roles in the nucleus rather than being targeted to the ribosome for translation. In keeping with this, hypoxia-induced NEAT1 was localized to the nucleus suggesting that NEAT1 plays a specific role in oxygen-sensitive regulation within cell nuclei (Figure 1i).

### NEAT1 is induced by hypoxia in solid tumors

**Figure 2:**
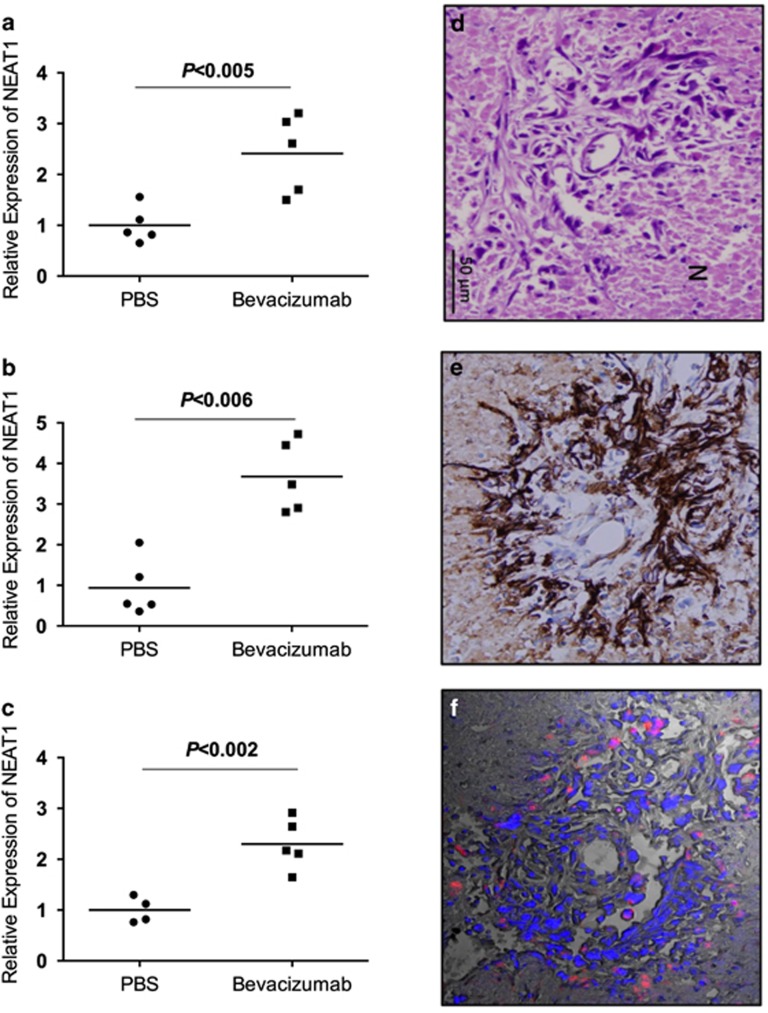
NEAT1 is induced by hypoxia in solid tumors. qPCR analysis of NEAT1 expression in (**a**) MCF-7, (**b**) MDA-MB-231 and (**c**) MDA-MB-468 tumor xenografts treated with the anti-angiogenesis agent, bevacizumab, or vehicle only showing increased NEAT1 levels following treatment with bevacizumab. Three independent experiments were analyzed on a minimum of 100 cells per replicate. (**d**) H&E staining, (**e**) immunohistochemistry for CA9 and (**f**) RNA-fluorescent *in situ* hybridization analysis for NEAT1 (red channel=NEAT1, blue channel=DAPI counterstain) in MDA-MB-231 tumor xenografts showing co-localization of NEAT1 and CA9 expression in a penumbra distant from a blood vessel.

To determine whether hypoxic induction of NEAT1 occurs more generally, we first surveyed a panel of estrogen receptor-positive (+) and -negative (−) and triple receptor negative breast cancer cell lines for hypoxia-dependent regulation of NEAT1. Though responses did vary, most cell lines manifest induction of NEAT1 by hypoxia, with 6 out of 11 cell lines, across all receptor sub-types, showing statistically significant hypoxic induction (Figure 1j). Three breast cancer cell lines, MCF-7, MDA-MB-231 and MDA-MB-468, were then injected into nude mice and grown as xenografts. The mice were treated with either vehicle control or the angiogenesis inhibitor, bevacizumab, to increase tumor hypoxia. Treatment with bevacizumab increased NEAT1 mRNA levels in xenografts derived from all three cell lines (Figures 2a and c). As a control, levels of CA9 mRNA (a known hypoxia-induced gene) were measured in the same tumors (Supplementary Figure 3A-C). Furthermore, examining MDA-MB-231 xenografts for CA9 by immunostaining and NEAT1 by RNA-fluorescent *in situ* hybridization confirmed co-localization of NEAT1 with CA9 in regions distant from tumor blood vessels (Figures 2d and e). Furthermore, NEAT1 is more highly expressed in the more hypoxic superficial regions of mouse gastric epithelium.^28^ Together with our findings, this indicates that NEAT1 is regulated by hypoxia, both *in vitro* and *in vivo* in solid tumors.

### Hypoxic induction of NEAT1 by HIF-2 induces the formation of nuclear paraspeckles

NEAT1 is the main architectural component required for the formation of paraspeckles, which form between 2 and 20 nuclear structures of approximately 360 nm in diameter in each mammalian cell. They were originally defined as foci rich in four RNA-binding proteins: RBM14, PSPC1, non-POU domain containing octamer binding protein (NONO, also called p54arb) and SFPQ. We therefore next determined the role of hypoxia-induced NEAT1 in the formation of nuclear paraspeckles.

**Figure 3:**
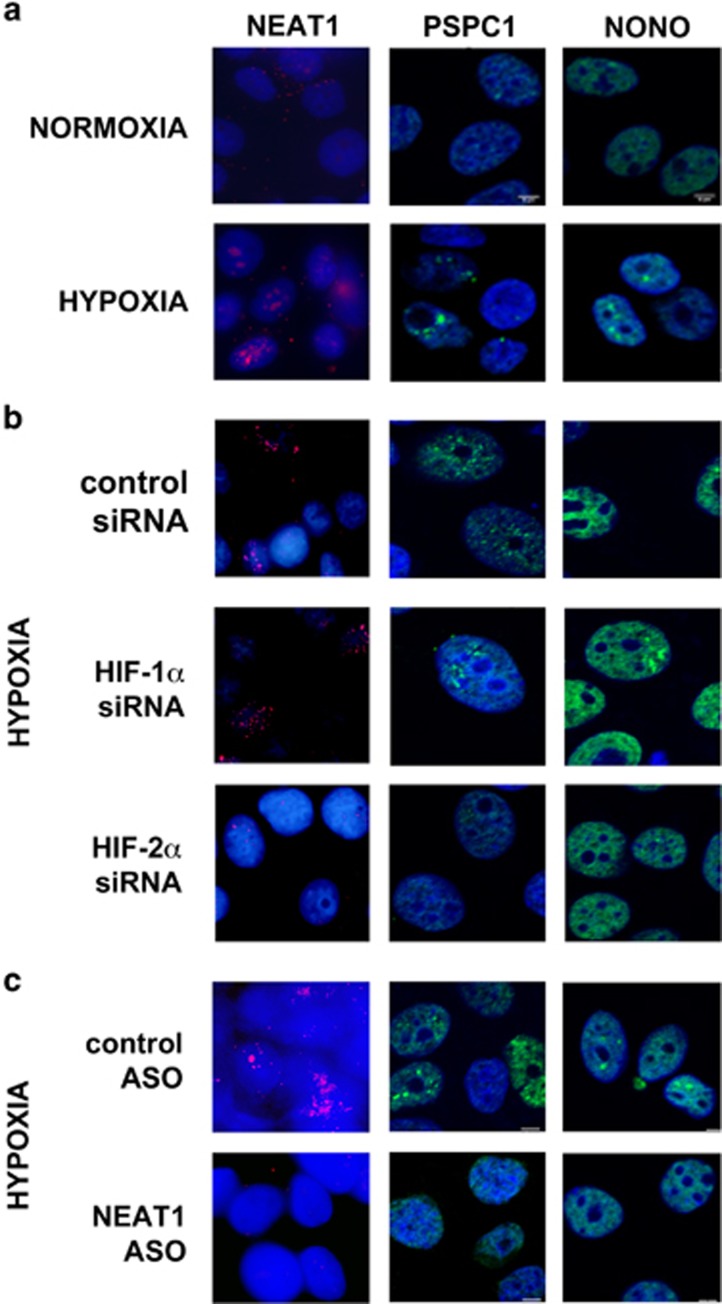
Hypoxic induction of NEAT1 by HIF-2 leads to the formation of nuclear paraspeckles. (**a**) RNA-fluorescent *in situ* hybridization for NEAT1 (red channel) showing hypoxic induction of NEAT1 in condensed nuclear structures in hypoxic MCF-7 cells. Immunofluorescence (green channel) for the paraspeckle proteins, PSPC1 and NONO (p54nrb), showing aggregation of nuclear paraspeckles in hypoxia. Cell nuclei were counterstained with DAPI (blue channel). (**b**) RNA-fluorescent *in situ* hybridization and immunofluorescence in hypoxic MCF-7 cells treated with control siRNA, HIF-1α siRNA or HIF-2α siRNA, showing depletion of hypoxia-induced NEAT1 and paraspeckles following suppression of HIF-2α. (**c**) Immunofluorescence of hypoxic MCF-7 cells treated with control or NEAT1 antisense oligonucleotides (ASO) showing depletion of hypoxia-induced paraspeckles following suppression of NEAT1.

We first determined the nuclear distribution of NEAT1 in MCF-7 cells, using RNA-fluorescent *in situ* hybridization. In normoxic cells, we observed low NEAT1 signal, with no or few, weakly staining foci in each nucleus. In hypoxia, NEAT1 was distributed in a punctate pattern in the nuclei of hypoxic cells consistent with its localization in nuclear paraspeckles and both the number and intensity of the foci was increased compared with normoxic cell nuclei (Figure 3a and Supplementary Figure 4). We then determined whether the nuclear distribution of the protein components of paraspeckles was affected by hypoxia. The total expression of the paraspeckle-associated proteins, PSPC1 and NONO, was unaffected by hypoxia (Supplementary Figure 5). However, the distribution of each protein within the nucleus was altered. Both proteins adopted a more punctate distribution in hypoxia indicating increased formation of nuclear paraspeckles in hypoxia (Figure 3a and Supplementary Figure 4).

As NEAT1 is induced by HIF-2 in hypoxia, we next determined the dependence of paraspeckle formation on HIF-1α and HIF-2α using both RNA-fluorescent *in situ* hybridization for NEAT1 and immunofluorescence for PSPC1 and NONO. Suppression of hypoxic HIF-2α by siRNA abrogated the aggregation of PSPC1 and NONO into paraspeckles (Figure 3b). Conversely, the suppression of HIF-1α had little or no effect on the nuclear distribution of PSPC1 or NONO.

Finally, although previous work in normoxia has shown that the formation of paraspeckles requires NEAT1,^15, 18, 19, 20^ we next determined whether their formation in hypoxia was also dependent on the hypoxic induction of NEAT1. Expression of NEAT1 was inhibited in MCF-7 cells using antisense oligonucleotides (ASOs) and suppression compared with control oligonucleotides was confirmed by qPCR (Supplementary Figure 6). Cells were then incubated in 1% hypoxia for 24 h. Suppression of NEAT1 inhibited the hypoxic formation of nuclear paraspeckles. Taken together, these data indicate that hypoxic induction of NEAT1 by HIF-2 leads to the formation of nuclear paraspeckles.

### Hypoxic induction of NEAT1 results in nuclear retention of F11R (JAM1) mRNA

The precise function of NEAT1 and nuclear paraspeckles remains unclear. However, paraspeckles are known to have roles both in the sequestration of paraspeckle-associated proteins, which depletes their levels in the nucleoplasm and in the nuclear retention of A-to-I-edited RNA transcripts, preventing their translation in cytoplasmic ribosomes. A-to-I editing is a form of post-transcriptional processing that is particularly promiscuous on Alu repeat elements, which form double-stranded RNA hairpins and are present in the extended 3′-UTRs of more than 300 genes. Recently, one such transcript, F11R, was shown to be retained in the nucleus in hypoxia.^29^ F11R also called junctional adhesion molecule 1 (JAM1) is a member of the immunoglobulin superfamily and is an important regulator of tight junction assembly in epithelia. We therefore examined the dependence of hypoxic F11R mRNA nuclear retention on NEAT1 induction.

**Figure 4:**
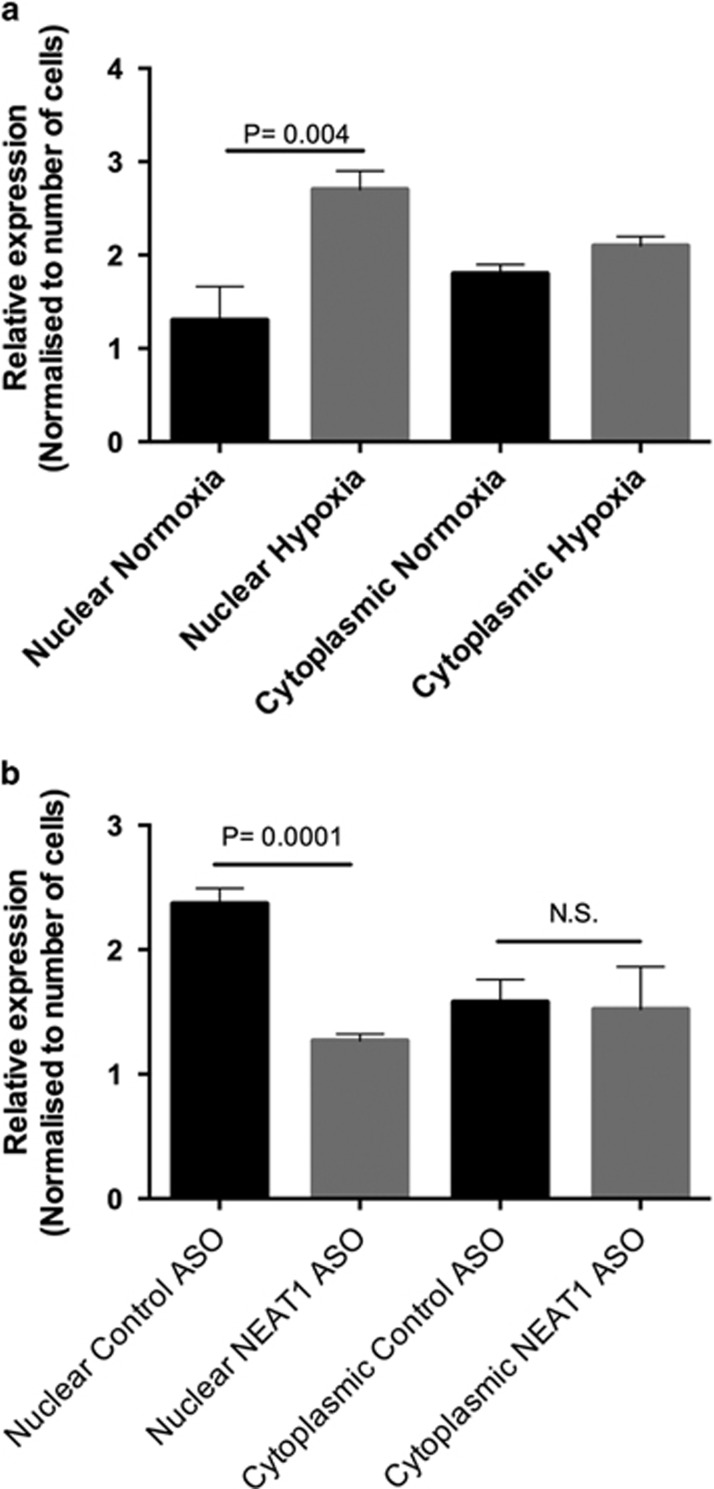
Hypoxic induction of NEAT1 results in nuclear retention of F11R (JAM1) mRNA. (**a**) qPCR analysis of nuclear and cytoplasmic fractions from MCF-7 cells incubated in normoxia or 1% hypoxia showing hypoxic induction of nuclear, but not cytoplasmic, F11R transcript. (**b**) qPCR analysis of nuclear and cytoplasmic fractions from hypoxic MCF-7 cells treated with either control or NEAT1 ASO showing the reduction of hypoxic F11R in the nucleus following suppression of NEAT1.

We first confirmed the hypoxia inducibility of nuclear F11R mRNA in MCF-7 cells. qPCR analysis of nuclear and cytoplasmic extracts from cells incubated under 1% hypoxia or normoxia for 24 h demonstrated approximately twofold induction in nuclear levels, with negligible induction of cytoplasmic levels of F11R mRNA (Figure 4a). Hypoxic MCF-7 cells were then pre-treated with either control or NEAT1 ASOs. Suppression of NEAT1 abrogated hypoxic induction of nuclear F11R, but had no effect on cytoplasmic levels (Figure 4b). Taken together this demonstrates that hypoxia-induced NEAT1 increases nuclear, but not cytoplasmic, levels of F11R mRNA indicating a role in nuclear retention of this mRNA.

### Hypoxic induction of NEAT1 accelerates tumor cell proliferation and inhibits apoptosis

Activation of hypoxia pathways and HIF in particular is associated with an aggressive tumor phenotype and poor clinical outcome in many types of cancer, including breast cancer. Although many HIF-dependent coding genes contribute to these properties, the involvement of HIF-regulated lncRNAs remains unclear. We therefore determined whether the hypoxic induction of NEAT1 influenced the behavior of breast cancer both *in vitro* and *in vivo*.

**Figure 5:**
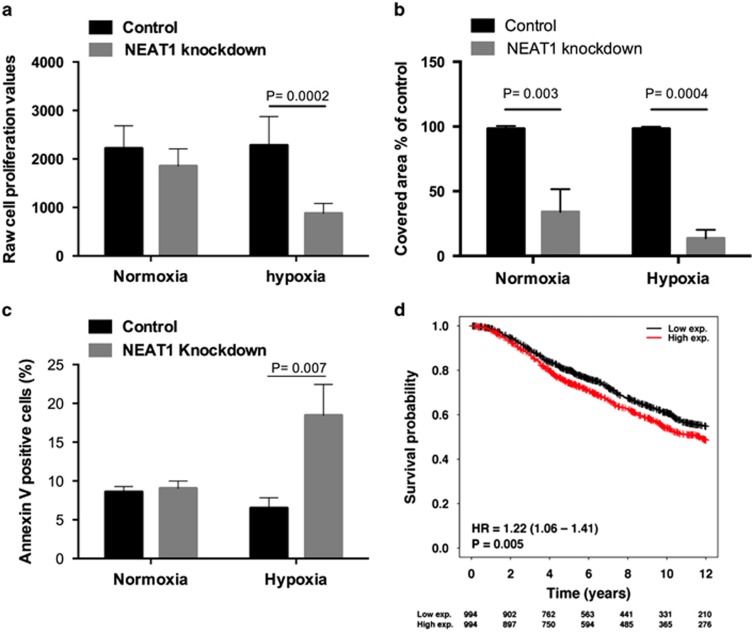
Hypoxic induction of NEAT1 accelerates tumor cell proliferation, inhibits apoptosis and is associated with adverse clinical outcome in patients with breast cancer. (**a**) Cell proliferation rates (normalized to control ASO), (**b**) colony formation rate and (**c**) annexin V staining for normoxic and hypoxic MCF-7 cells treated with either control ASO or NEAT1 ASO showing reduced proliferation, reduced colony formation and increased apoptosis following NEAT1 depletion. Each experiment was performed with three biological replicates (*P<0.05, **P⩽0.009, ***P⩽0.0009, Student's *t*-test). (**d**) Kaplan–Meier plot for 2000 breast cancer patients from the Metabric trial stratified according to expression of NEAT1 mRNA (above versus below median) showing that high tumor NEAT1 levels are associated with increased patient mortality.

We first examined the effects of NEAT1 on cell proliferation and colony formation in normoxia and in hypoxia. MCF-7 cells were pre-treated with either control or NEAT1 ASOs for 48 h prior to incubation in either normoxia or 1% hypoxia for a further 24 h and cell proliferation rates were then determined. Suppression of NEAT1 reduced cell proliferation rates in both normoxia and hypoxia (Figure 5a). However, the effect of NEAT1 suppression was greater in hypoxia indicating that NEAT1 has a larger effect on cell proliferation in hypoxia than it does in normoxia. Suppression of NEAT1 also reduced colony formation in both normoxia and hypoxia (Figure 5b). Again the effect of NEAT1 suppression was larger in hypoxia, when NEAT1 levels are higher, than it was in normoxia. Similar results were observed in ZR-75-1 cells (Supplementary Figures 7A and B).

Next, we looked at the effects of NEAT1 on apoptosis in normoxia and in hypoxia. MCF-7 cells were again pre-treated with control or NEAT1 ASOs prior to incubation in normoxia or 1% hypoxia and apoptosis was assessed by annexin V staining. Hypoxia led to a small, although non-significant fall in apoptosis in the control-treated cells. However, following suppression of NEAT1, hypoxia dramatically increased apoptosis, indicating that NEAT1 was inhibiting apoptosis in hypoxic cells (Figure 5c and Supplementary Figure 7C).

### High tumor NEAT1 expression is associated with poor patient prognosis in breast cancer

Finally, the expression levels of a number of lncRNAs are associated with clinical outcome and pathological features in a variety of cancers. To evaluate the clinical relevance of NEAT1 in breast cancer, we determined whether tumor NEAT1 expression levels were correlated with patient prognosis. Using expression data derived from a cohort of 2000 patients with breast cancer,^30^ patients were categorized according to NEAT1 expression. Kaplan–Meier analysis of overall mortality (Figure 5d) revealed that patients with high tumor NEAT1 expression have a significantly poorer outcome when compared with patients with low tumor NEAT1 expression (*P*=0.005, hazard ratio 1.22 95% confidence interval=1.06–1.41). Multivariate analysis of NEAT1 expression, adjusted for clinicopathological features known to correlate with outcome (age, tumor size, grade, stage and node status), showed that NEAT1 remains significantly associated with poor outcome after adjustment for age (*P*=0.013), tumor size (*P*=0.008), grade (*P*=0.017) and nodes status (*P*=0.007). This indicates that tumor NEAT1 expression is an independent prognostic marker in breast cancer patients, at least with respect to these variables.

## Discussion

In addition to regulating the coding transcriptome, it is now becoming apparent that hypoxic transcriptional pathways orchestrated by HIF also control the expression of non-coding regulatory transcripts, particularly lncRNAs.^5^ However, to date, little is known about the biological function of many of these lncRNAs and how they impact upon the regulation of downstream genes in hypoxia. Here, we show that one of the most abundant and highly upregulated lncRNAs, NEAT1, induces the formation of nuclear paraspeckles in hypoxia in a manner that depends upon the HIF-2 transcription factor. This indicates a novel function for HIF pathways in the regulation of nuclear structure in hypoxia. Furthermore, electron microscopy studies,^31, 32^ immunostaining of nuclear proteins^33^ and the recognition that chromatin-modifying enzymes are oxygen- and HIF-dependent^34^ all suggest a more widespread role for hypoxia in regulating nuclear architecture. Other lncRNAs may play a role in this. MALAT1, which associates with nuclear speckles, is syntenic with NEAT1 and is alone in sharing an unusual 3′-end, indicating that the two lncRNAs may share similar functions. In a recent pangenomic analysis,^5^ MALAT1 was also regulated by hypoxia and had a HIF-binding site close to its promoter. Whether this hypoxic induction of MALAT1 alters the formation of nuclear speckles remains to be determined.

To date, little is known about the regulation of NEAT1 or of paraspeckles. NEAT1 is activated during differentiation of embryonic stem cells and is widely expressed in mammalian cells.^18^ In addition to hypoxia, NEAT1 can also be activated by various viral infections including Japanese encephalitis, rabies and HIV,^35^ and is frequently upregulated in many types of cancer. Other cellular stresses, in particular proteasomal inhibitors, can also induce NEAT1 mRNA^21^ and this may, at least in part, be mediated by the stabilization of HIF-2α by these drugs.

The biological functions of paraspeckles are currently poorly understood, but they are thought to have regulatory roles in gene expression, either through the sequestration of transcriptionally active proteins or through the retention of A-to-I-edited RNA transcripts or potentially both mechanisms.^23^ Recently, A-to-I-edited F11R transcripts were also shown to be retained in the nucleus in hypoxia in association with p54nrb.^29^ We also observed retention of F11R mRNA in the nucleus in hypoxia and showed that this requires the hypoxic induction of NEAT1. Interestingly, the main function of another retained transcript, Ctn, was to provide a reservoir of RNA that could be released into the cytoplasm in response to stress signals.^29^ Therefore, although we did not observe any alteration in cytoplasmic F11R mRNA in hypoxia, we hypothesize that this may provide a pool of F11R mRNA that can be released into the cytoplasm upon re-oxygenation. This is analogous to the release of RNA transcripts from cytoplasmic stress granules following their depolymerization upon re-oxygenation of hypoxic cells.^36^ However, whether hypoxic retention of RNAs in nuclear paraspeckles is a general phenomenon, or is restricted to a few specific transcripts remains to be determined.

More recently, paraspeckles have been shown to affect gene transcription either through the sequestration of transcriptional enhancers or transcriptional repressors.^21, 22^ Notably, many HIF-regulated genes do not have neighboring HIF-binding sites suggesting that they may be controlled by other transcription factors that are themselves regulated by HIF. This is particularly the case for genes that are downregulated by the HIF pathway.^37, 38^ The HIF-dependent induction of paraspeckles leading to the sequestration of specific transcription factors may provide one such mechanism for this indirect gene regulation by HIF.

Activation of HIF pathways, particularly HIF-2, is associated with aggressive tumor behavior and poor patient prognosis across many types of cancer including breast cancer.^39, 40, 41, 42, 43^ Although upregulation of many protein-coding genes has a major role in this phenotype, the expression of many lncRNAs also correlates with adverse prognosis in cancer and the extent to which these contribute to the hypoxic tumor phenotype remains unclear. Here, we show that NEAT1 accelerates cell proliferation, promotes clonogenic survival and inhibits apoptosis in hypoxic breast cancer cells. This is consistent with previous findings in which NEAT1−/− mouse embryonic fibroblasts have been found to be more sensitive to apoptotic stimuli than wild-type mouse embryonic fibroblasts.^21^ In addition, high tumor NEAT1 expression correlates with poor survival in patients with breast cancer. Taken together, these results indicate that in addition to the protein-coding transcriptional response, hypoxic induction of NEAT1 lncRNA also contributes to the aggressive phenotype seen in hypoxic tumors.

## Materials and methods

### Cell culture and HIF knockdown

Human breast cancer cell lines were obtained from the American Type Culture Collection (ATCC, Manassas, VA, USA). Cells were incubated for 24 h in an *In vivo2* Hypoxia Work Station (Ruskinn Technology Ltd, Bridgend, UK) in an atmosphere of either normoxia (21% oxygen) or hypoxia (1% oxygen). HIF-1α and/or HIF-2α subunits were suppressed as previously described.^44^

### Chromatin Immunoprecipitation

This was performed as described previously.^38^

### Tumor xenografts

Xenograft experiments were performed in female BALB/c nunu (MCF-7 cells) or BALB/c SCID (MDA-MB-231 and MDA-MB-468 cells) mice. A total of 2.5 × 10^6^ (MCF-7) cells or 10 × 10^6^ (MDA-MB-231 or MDA-MB-468) cells were injected into mammary fat pads in equal volumes of Matrigel (BD Biosciences, Oxford, UK). Mice injected with MCF-7 cells had estrogen (5 μg/ml) added to their drinking water. Once tumors reached 150 mm^3^, mice received either intraperitoneal bevacizumab (10 mg/kg every 3 days) or vehicle control.

### Total RNA isolation

Total RNA was isolated using the mirVana miRNA Isolation Kit (Ambion—Life Technologies, Paisley, UK) and treated with DNase I (TURBO DNA-free, Ambion—Life Technologies). Fractionated nuclear and cytoplasmic RNAs were isolated using PARIS Protein and RNA Isolation kit (Ambion—Life Technologies).

### Reverse transcription and qPCR

cDNA was synthesized using SuperScript II Reverse Transcriptase (Invitrogen-Life Technologies, Paisley, UK). qPCR was performed using IQ SYBR Green Mix (Bio-Rad, Hemel Hempstead, UK) on the CFX96 Real-Time System (Bio-Rad) and normalized to RPL11 (60S ribosomal protein L11). All experiments were performed using three biological replicates. Primer sequences used for qPCR assays are given in Supplementary Table 1.

### Transfection of ASOs

NEAT1 and scrambled control ASOs (Integrated DNA Technologies, Coralville, IA, USA) were transfected using Lipofectamine RNAiMAX reagent (Invitrogen-Life Technologies) with two transfections carried out 24 h apart. ASO sequences are given in Supplementary Table 2.

### Immunofluorescence

Cells were fixed with 4% paraformaldehyde (Thermo Fisher Scientific, Cramlington, UK), permeabilized with 0.2% Triton X-100 (T8532, Sigma-Aldrich, Dorset, UK), blocked with 1% bovine serum albumin (Sigma-Aldrich) and incubated overnight with antibodies to PSPC1 (sc-374181, Santa Cruz Biotechnology, Dallas, TX, USA) or NONO (611279, BD Biosciences) followed by fluorescent 488-secondary antibody (Life Technologies, Paisley, UK) before mounting using DAPI Fluoromount G (Southern Biotech, Birmingham, AL, USA). Images were taken using a LSM 510 META confocal microscope (Zeiss, Oberkochen, Germany).

### Immunohistochemistry for xenografts

Breast cancer xenograft morphology was assessed using H & E staining. Immunohistochemistry for CA9 (M75 mouse monoclonal antibody) was performed as previously described.^45^ Slides were quantitated by color deconvolution in ImageJ.^46^

### RNA-fluorescent *in situ* hybridization

Cells were seeded onto circular coverslips in 24-well dishes and incubated in normoxia or hypoxia. Xenograft tissues, treated with PBS or bevacizumab, were fixed in formalin, and embedded in paraffin. Slides were prepared using the QuantiGene ViewRNA ISH Cell or Tissue Kit (Affymetrix, Santa Clara, CA, USA) using a NEAT1 probe (VA1-12621-01, Affymetrix). Cell nuclei were counterstained with DAPI. Slides were examined for epifluorescence using an Olympus BX60 microscope equipped with a Sensys CCD camera (Photometrics, Tucson, AZ, USA) and analyzed with Genus Cytovision 7.1 software (Leica Microsystems, Milton Keynes, UK).

### Annexin V apoptosis assay

Cells were collected, stained for Annexin V (Life Technologies), counterstained with propidium iodide and analyzed by CyAn ADP FACS analyzer (Beckman Coulter, High Wycombe, UK).

### Clonogenic assay

Clonogenic assays were performed as previously described^47^ and quantified using ImageJ software (http://imagej.nih.gov/ij/).

### Cell proliferation assay

Cell proliferation was measured using the CyQUANT NF Cell Proliferation Assay Kit (Invitrogen-Life Technologies).

### Statistical analysis

Statistical analyses were performed in R (http://www.R-project.org) using two-tailed *t*-tests or one-way analysis of variance with Dunnett's or Bonferroni post-test as appropriate.
