# Supplementary material for: Tumor hypoxia induces nuclear paraspeckle formation through HIF-2α dependent transcriptional activation of NEAT1 leading to cancer cell survival
Source: Oncogene. 2014 Nov 24;34(34):4482–90. doi: 10.1038/onc.2014.378 (PMC4430310; doi:10.1038/onc.2014.378)

## Supplemental Figure Legends

**Supplemental Figure 1. Hypoxic and DMOG dependent induction of NEAT1 in MCF-7 and ZR-75-1 cells.** qPCR analysis of relative NEAT1 expression in (A) MCF-7 and (B) ZR-75-1 cells after the indicated duration of incubation in 1% hypoxia, normalized to normoxic levels. ANOVA one-way analysis of variance with post-hoc analysis using Dunnett's test (**p<0.01, ***p<0.001). qPCR analysis of CA9 and NEAT1 expression in (C) MCF-7 and (D) ZR-75-1 cells treated with 0.5 mM dimethyloxalylglycine (DMOG) for 24 hours, compared to vehicle alone controls (**p<0.01, ***p<0.001, Student's t-test).

**Supplemental Figure 2. Suppression of HIF-α isoforms in hypoxia by siRNA.** Immunoblot analysis of HIF-1α and HIF-2α levels in hypoxic MCF-7 cells pretreated with siRNA against the designated isoform.

**Supplemental Figure 3. Bevacizumab induces expression of the hypoxia-responsive gene, CA9 in solid tumors.** qPCR analysis of CA9 expression in (A) MCF-7, (B) MDA-MB-231 and (C) MDA-MB-468 tumor xenografts treated with the anti-angiogenesis agent, Bevacizumab or vehicle only showing increased CA9 levels following treatment with Bevacizumab comparable to those seen for NEAT1.

**Supplemental Figure 4. Hypoxic induction of NEAT1 and nuclear paraspeckles in ZR-75-1 cells.** (A) RNA-FISH for NEAT1 (red channel) showing

hypoxic induction of NEAT1 in condensed nuclear structures in hypoxic MCF-7 cells. Immunofluorescence (green channel) for the paraspeckle proteins, PSPC1 and NONO (p54nrb), showing aggregation of nuclear paraspeckles in hypoxia. Cell nuclei were counterstained with DAPI (blue channel).

**Supplemental Figure 5. Total expression of PSPC1 and NONO protein was unaffected by hypoxia.** Immunoblot analysis of PSPC1, NONO and ACTB protein in MCF-7 cells incubated in either normoxia (N) or 1% hypoxia (H) for 24 hours.

**Supplemental Figure 6. Suppression of NEAT1 expression by antisense oligonucleotides (ASOs).** qPCR analysis demonstrates that NEAT1 expression is significantly suppressed (>85%) in MCF-7 and ZR-75-1 cells grown in hypoxic conditions, using two different ASOs.

**Supplemental Figure 7. Hypoxic induction of NEAT1 accelerates tumor cell proliferation, inhibits apoptosis in ZR-75-1 cells.** (A) Cell proliferation rates (normalized to control ASO), (B) Colony formation rate and (C) Annexin V staining for normoxic and hypoxic ZR-75-1 cells treated with either control ASO or NEAT1 ASO showing reduced proliferation, reduced colony formation and increased apoptosis following NEAT1 depletion. Each experiment was performed with three biological replicates.

**Supplementary Table 1. qPCR primer sequences**

| Primer name | Sequence 5'-3' |
|---|---|
| NEAT1-1 FW | CCAGTTTTCCGAGAACCAAA |
| NEAT1-1 RV | ATGCTGATCTGCTGCGTATG |
| NEAT1-2 FW | CTAGAGGCTCGCATTGTGTG |
| NEAT1-2 RV | GCCCACACGAAACCTTACAT |
| F11R FW | GCCCGAAGTGAAGGAGAATTC |
| F11R RV | CAGATGATAGGCGGTGAGCC |
| NEAT1 ChIP FW | GAACCACCGCCCGAAAGT |
| NEAT1 ChIP RV | CGGCAGGACATCTGGAAAAT |
| ACTG ChIP FW | AGATGTGGATTAGCAAGCAGG |
| ACTG ChIP RV | GCTTATTCCAGTTTCGTGAGGC |
| NDRG1 ChIP FW | TCCCTCCCAATCTCTCTCTTCTT |
| NDRG1 ChIP RV | CACCATCAGCACAGCAAACTAC |
| EGLN3 ChIP FW | CCAGTCACCAGAGAAATGTT |
| EGLN3 ChIP RV | GCGTCTTGATGTCCTTATCCCT |

**Supplementary Table 2. Sequences of NEAT1 ASO**

| ASO | Sequences |
|---|---|
| NEAT1 ASO1 | 5'-mC-mC-mC-mU-mU-C-T-C-C-T-A-G-T-A-A-mU-mC-mU-mG-mC-3' |
| NEAT1 ASO2 | 5'-mC-mC-mC-mU-mC-T-A-G-T-C-T-T-G-G-C-mU-mC-mA-mU-mU-3' |
| Scrambled control ASO | 5'-mU-mA-mU-mC-mU-G-C-A-C-T-T-C-T-C-T-mCmA-mC-mA-mC-3' |

**Supplemental Figure 1**

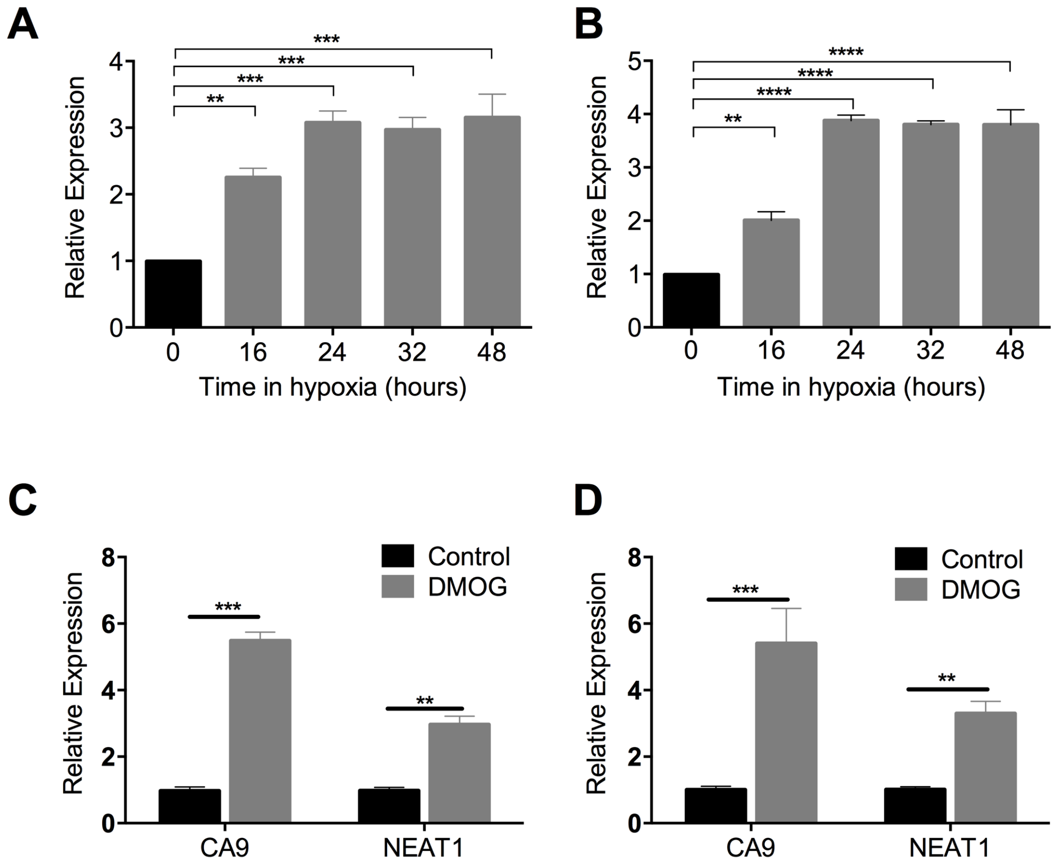

**Supplemental Figure 2**

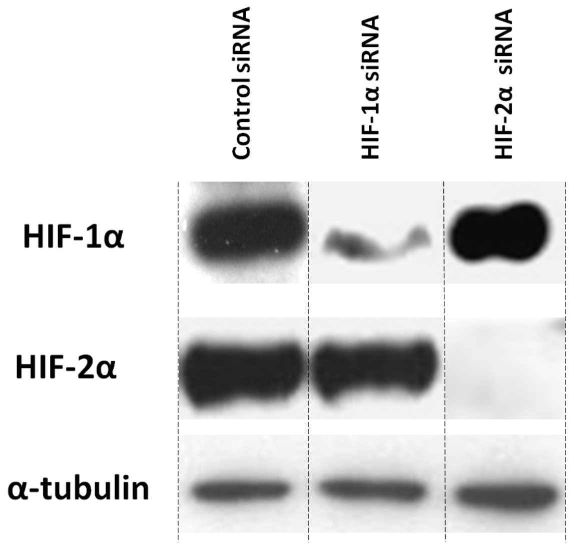

**Supplemental Figure 3**

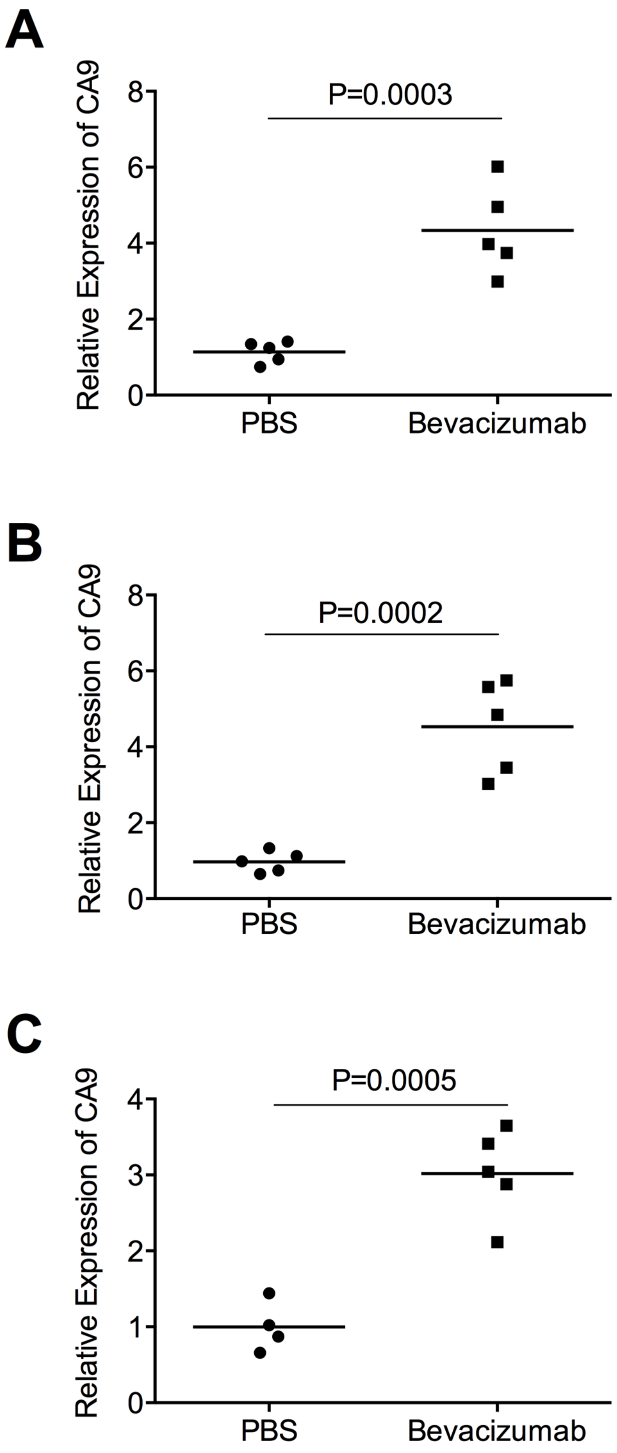

**Supplemental Figure 4**

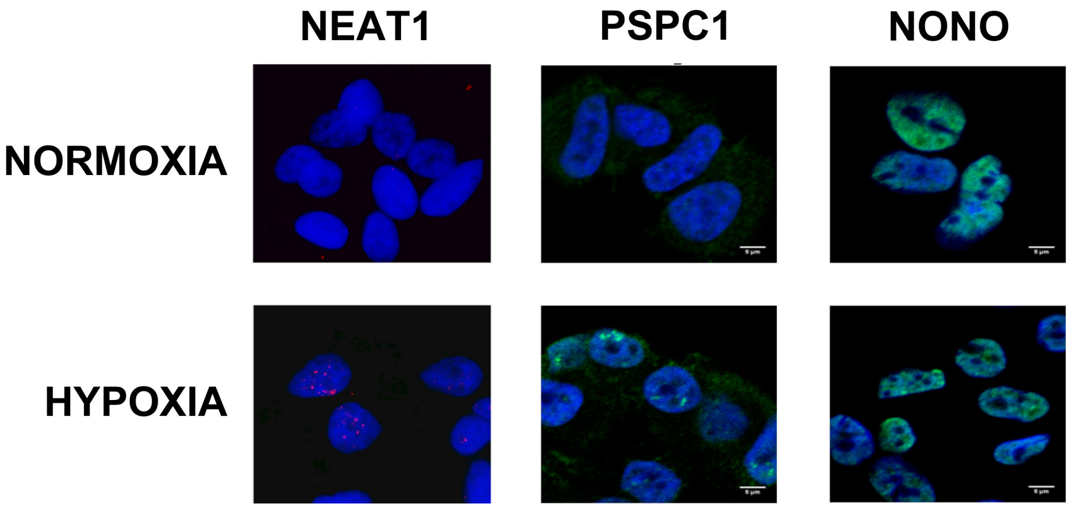

**Supplemental Figure 5**

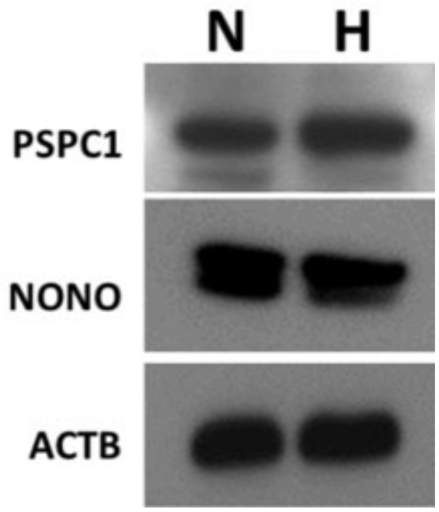

**Supplemental Figure 6**

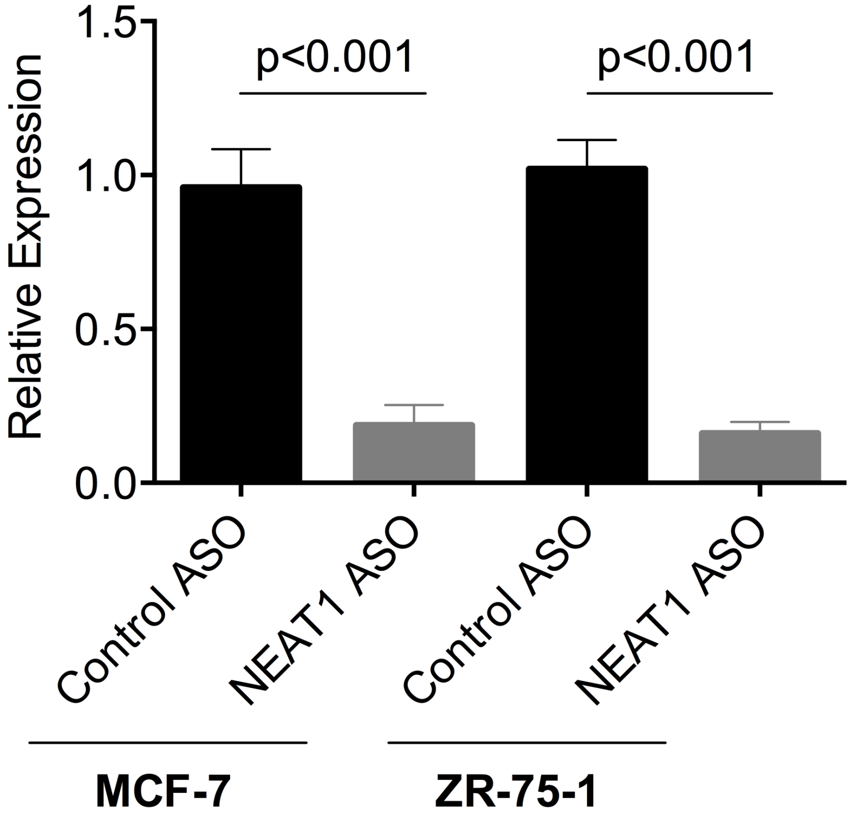

**Supplemental Figure 7**

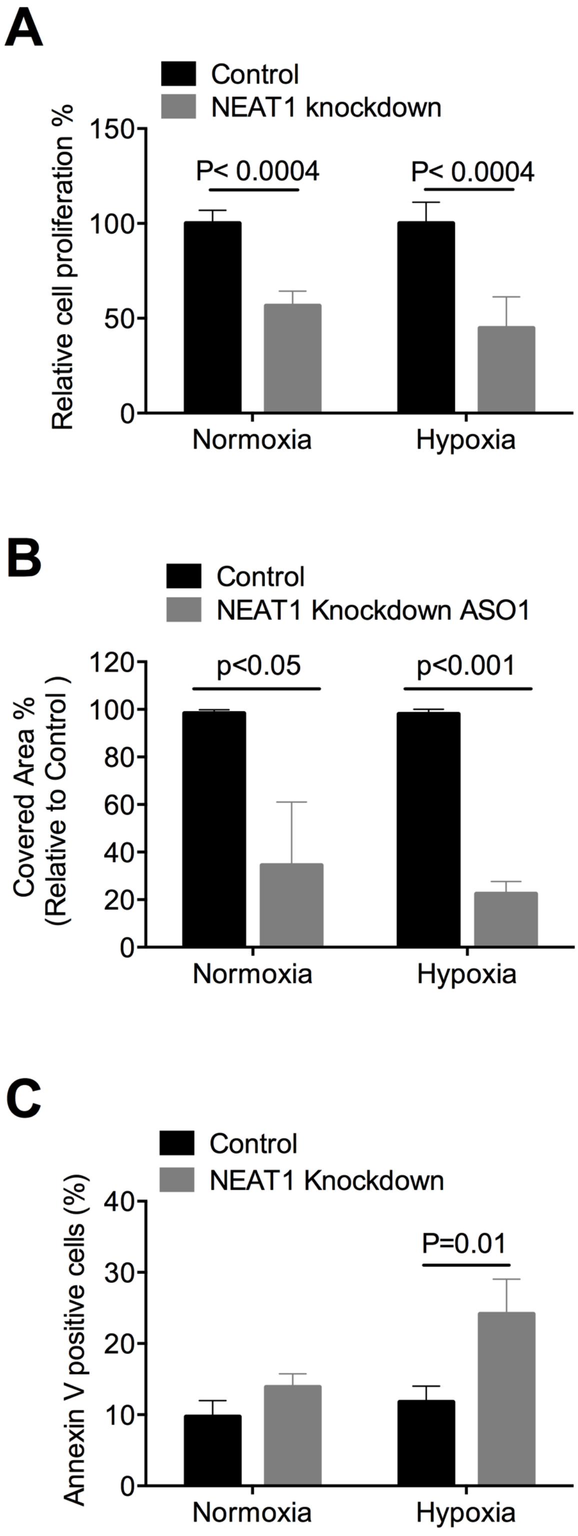

Supplement: Supplementary Information [file onc2014378x1.pdf]
